# The longitudinal relationship between economic and social stressors, emotion dysregulation and mental health among refugees in protracted displacement

**DOI:** 10.1017/S2045796026100493

**Published:** 2026-03-27

**Authors:** Philippa Specker, Gülşah Kurt, Belinda J Liddell, David Keegan, Randy Nandyatama, Atika Yuanita, Rizka Argadianti Rachmah, Joel Hoffman, Shraddha Kashyap, Diah Tricesaria, Mitra Khakbaz, Zico Pestalozzi, Angela Nickerson

**Affiliations:** 1 School of Psychology, University of New South Wales, Sydney, NSW, Australia; 2 School of Psychological Sciences, The University of Newcastle, Newcastle, NSW, Australia; 3 School of Social Work, Excelsia University College, Macquarie Park, NSW, Australia; 4 HOST International, Parramatta, NSW, Australia; 5 Department of International Relations, Universitas Gadjah Mada, Yogyakarta, Indonesia; 6 SUAKA, Indonesian Civil Society Network for Refugee Rights Protection, Jakarta Pusat, Indonesia; 7 Sydney School of Medicine, University of Sydney, Sydney, NSW, Australia; 8 Bilya Marlee School of Indigenous Studies, The University of Western Australia, Perth, WA, Australia; 9 School of Social Sciences, Monash University, Melbourne, VIC, Australia

**Keywords:** emotion regulation, mental health, refugees, social and economic stressors

## Abstract

**Aims:**

Trauma-related psychopathology is markedly elevated among refugee populations, particularly those living in sustained displacement. While economic, social and psychological factors have been linked to the deterioration of mental health following trauma and displacement, these factors have rarely been investigated concurrently and longitudinally. Consequently, there is little information on the potential longitudinal mechanisms driving mental ill-health in displacement settings. This study explored the temporal association between economic stressors, social stressors, emotion dysregulation and psychopathology in 1,235 refugees displaced in Indonesia.

**Methods:**

Refugee participants from Farsi, Dari, Arabic, Somali and English-speaking backgrounds completed an online survey at four timepoints, 6 months apart. Factors of interest were measured using validated instruments including the Patient Health Questionnaire (to assess depressive symptoms), Posttraumatic Diagnostic Scale (to assess posttraumatic stress [PTS] symptoms), Post-Migration Living Difficulties Checklist (to index economic and social stressors) and Difficulties in Emotion Regulation Scale (to assess emotion dysregulation).

**Results:**

Random-intercept cross-lagged panel analysis revealed that economic stressors and emotion dysregulation were central to the longitudinal course of trauma-related psychopathology. Specifically, economic stressors were associated with subsequent increases in PTS symptoms (*B* = 0.07, *p* = 0.047), depressive symptoms (*B* = 0.17, *p* < .001) and social stressors (*B* = 0.28, *p* < .001), while emotion dysregulation was antecedent to increases in PTS (*B* = 0.16, *p* < .001), depression symptoms (*B* = 0.13, *p* < .001), and social stressors (*B* = 0.10, *p* = .017). Additionally, depression was associated with subsequent increases in economic stressors (*B* = 0.18, *p* = .001) and social stressors were associated with subsequent increases in economic stressors (*B* = 0.12, *p* = .037).

**Conclusions:**

The current study identified both economic stressors and emotion dysregulation as the main drivers of psychopathology for refugees. This indicates that both the structural barriers encountered in the environment and one’s internal capacity have a substantial impact on wellbeing. These findings highlight that alongside psychological interventions, policy changes that facilitate economic empowerment are critically, and equally, important.

People from refugee^1^ backgrounds represent one of the largest at-risk groups for developing trauma-related psychopathology. Elevated levels of posttraumatic stress disorder (PTSD) and depression have been consistently observed in refugees, with an estimated prevalence of 31.46% and 31.5%, respectively (Blackmore *et al.*, 2020). At the same time, the number of refugees and other forcibly displaced people has surpassed 120 million globally, with 75% residing in low- and middle-income countries (LMICs) (UNHCR, 2024b). Governments and NGOs in LMICs often have limited resources to address the public health needs of displaced populations (Lindert *et al.*, 2016). This situation is further complicated by a lack of information to guide policymakers and organisations in how best to allocate and expend their limited resources, such as whether to prioritise psychological treatments, psychosocial interventions, structural and economic support, or indeed, some combination of these approaches (Miller *et al.*, 2021). To meet this challenge there is a need to pinpoint the factors that are most salient in driving the symptoms of psychopathology among refugees living in protracted displacement.

Multiple theoretical models foreground the importance of considering both psychological and environmental factors as potential mechanisms underpinning refugee mental health (Kashyap *et al.*, 2021; Miller and Rasmussen, 2024). In particular, the Psychological Interaction with Environment (PIE) Matrix Model argues that environmental stressors interact with internal psychological processes to shape the mental health of refugees (Kashyap *et al.*, 2021). Despite this, much longitudinal research on trauma-related psychopathology has focused solely on individual-level psychological factors. Environmental stressors, however, may be particularly influential determinants of mental health for refugees displaced in LMICs who face harsh environmental conditions. For example, displaced refugees contend with diffuse economic stressors, including poverty, housing insecurity, lack of work rights and few formal supports (Miller *et al.*, 2008; Rasmussen *et al.*, 2010). Similarly, protracted displacement also disrupts social networks. Social stressors including isolation, forced separation from family and loneliness are prevalent issues among refugee populations and have been associated with poorer mental health (Liddell *et al.*, 2021; Kurt *et al.*, 2023). Miller and Rasmussen (2024), in their ecological model of mental health, argue that these chronic and uncontrollable stressors play an insidious role in eroding the mental health of conflict-affected populations. In particular, economic stressors that give rise to financial insecurity are especially salient contextual stressors; directly increasing one’s susceptibility to psychopathology (Torlinska *et al.*, 2020). Similarly, social stressors, including isolation and reduced social support, have been robustly associated with deteriorations in refugee mental health (Wachter *et al.*, 2022). Moreover, economic and social stressors are also inter-related, with poverty and social inequality evidencing a mutually reinforcing relationship, which can further compound mental health difficulties (Butterworth *et al.*, 2012; Torlinska *et al.*, 2020).

To date, much of the extant longitudinal research with refugee samples has been conducted in high-income countries. For example, regarding economic stressors, Torlinska *et al.* (2020) found that refugees were more than twice as likely to have PTSD if they also skipped meals due to a shortage of money, while O’Donnell *et al.* (2020) found a reciprocal, mutually reinforcing, longitudinal relationship between financial hardship and psychological distress. Similarly, regarding social stressors, Nguyen *et al.* (2024) found that social integration stressors (e.g., discrimination, lower sense of belonging, loneliness and lower English proficiency) were linked to increases in psychological distress overtime, while Chen *et al.* (2017) found loneliness to be driving the relationship between past trauma and current PTSD.

Concurrently, emotion dysregulation – defined as impairments in one’s capacity to monitor and modulate emotional responses (Gratz and Roemer, 2004) – has been robustly identified as an important transdiagnostic mechanism (Hofmann *et al.*, 2012; Tull *et al.*, 2020). Emotion dysregulation may be especially relevant for refugees, whose experiences of trauma, displacement and stressors give rise to negative emotions. Indeed, cross-sectional, experimental and treatment research with refugees in high-income countries has highlighted a link between emotion dysregulation and psychopathology (Specker and Nickerson, 2019, 2022; Koch *et al.*, 2020). In particular, a longitudinal study found that emotion dysregulation drove subsequent increases in PTS symptoms overtime (Specker *et al.*, 2024).

Despite important advances in our understanding of the diverse factors influencing refugee mental health, two important gaps in the evidence-base remain. First, economic stressors, social stressors and emotion dysregulation have rarely been investigated concurrently. This is problematic, as such factors likely interact to precipitate and maintain psychopathology (Kashyap *et al.*, 2021; Miller and Rasmussen, 2024). For example, the protracted stressors encountered by displaced refugees, such as economic hardship and social isolation, are notable sources of distress that can deplete internal coping resources (such as one’s emotion regulation capacity) and, in turn, exacerbate psychological symptoms. Second, our existing evidence-base has been dominated by research conducted in high-income countries, despite the fact that 75% of the global population of refugees reside in LMICs where environmental stressors are especially pronounced (UNHCR, 2024b). In such settings, understanding how multiple factors may differentially influence psychopathology is an essential precursor to determining the most effective way to support displaced refugees.

To address extant gaps in our knowledge, the current study sought to investigate the longitudinal relationship between economic and social stressors, emotion dysregulation, and PTS and depression symptoms among a large cohort of refugees displaced in Indonesia. Indonesia is a middle-income country where over 12,000 refugees reside for protracted periods (often exceeding 10 years), awaiting a permanent resettlement place in a third country (UNHCR, 2024a). In Indonesia, refugees do not have work rights or social security and have very limited access to government-funded support services. There are no formal refugee camps in Indonesia, instead, refugees reside in urban settings across multiple cities, where many encounter significant economic and social stressors including poverty, isolation and homelessness (UNHCR, 2024a). In line with prior longitudinal research on economic stressors, social stressors and emotion dysregulation (Chen *et al.*, 2017; O’Donnell *et al.*, 2020; Torlinska *et al.*, 2020; Nguyen *et al.*, 2024; Specker *et al.*, 2024), we anticipated that all constructs would be temporally associated with PTS and depression symptoms. Specifically, we hypothesised a bi-directional relationship between economic stressors and psychopathology, as well as social stressors and psychopathology, and that emotion dysregulation would be antecedent to increases in psychopathology.

## Method

### Participants

Participants were 1,235 refugees displaced in Indonesia. Participants were predominantly recruited via social media and snowball sampling. Eligibility criteria were: having a refugee background, being at least 18 years old, having arrived in Indonesia from January 2013 onwards and being literate in one of the available survey languages (Arabic, Farsi, Dari, Somali or English). The survey languages represented more than 80% of the languages spoken by the refugee population in Indonesia at the time of the study’s inception (UNHCR, 2018).

Of the 1,235 participants that completed the first timepoint (T1), 961 participants completed T2, 772 participants completed T3 and 748 participants completed T4. The overall retention rate was 61%. Correcting for multiple comparisons (Bonferroni, *α* = 0.05/13 = 0.004) there were no significant differences between participants who completed all four timepoints and those who did not in terms of baseline PTS symptoms (mean = 0.97 vs 0.96, *t*(1023) = 0.20, *p* = 0.842), depression symptoms (mean = 1.37 vs 1.27, *t*(1166) = 2.18, *p* = 0.034), emotion dysregulation (mean = 39.77 vs 39.67, *t*(1068) = 0.96, *p* = 0.923), social stressors (mean = 2.50 vs 2.34, *t*(835) = 2.63, *p* = 0.009), economic stressors (mean = 3.36 vs 3.18, *t*(849) = 2.63, *p* = 0.009), trauma exposure (mean = 7.86 vs 5.57, *t*(1010) = 0.93, *p* = 0.353), age (mean = 30.64 vs 30.42, *t*(1230) = 0.42, *p* = 0.679) or gender (74.05% males vs 69.33% males, *χ^2^*(1) = 3.38, *p* = 0.066). However, minor differences in some baseline demographic factors were present.^2^

### Measures

Measures were administered in Arabic, Farsi, Dari, Somali or English. Non-English measures underwent a rigorous translation process, starting with the translation and blind back translation by accredited translators, followed by pilot testing with refugees from each language group (and with varying educational backgrounds). Finally, feedback was integrated into the final versions to maximise the clarity and appropriateness of the measures.

**Demographic characteristics.** Baseline demographic information (age, gender, years lived in Indonesia, survey language and exposure to potentially traumatic events [PTEs]) was collected.

**Psychopathology.** PTS symptoms were measured using 20 items; 16 items from the Posttraumatic Diagnostic Scale for DSM-IV (PDS; Foa *et al.*, 1997) and 4 items to index the additions to the DSM 5 criteria (persistent negative beliefs, distorted blame, negative emotional state and risk-taking). Depressive symptoms were measured using the 8-item version of the Patient Health Questionnaire (PHQ-8; Kroenke *et al.*, 2009). Probable PTSD and probable depression at baseline were calculated using DSM-5 diagnostic algorithms. For the present analysis, mean scores for PTS and depression symptom severity were computed for each timepoint. Internal consistency was excellent across all timepoints (PTS: *α*’s = .95 to .97, depression: *α*’s = .89 to .93).

**Economic and social stressors.** Separate indexes of social and economic stressors were derived from the Post-Migration Living Difficulties Checklist (Silove *et al.*, 1998; Steel *et al.*, 1999). The present study followed the conceptualisation by Li *et al.* (2016), which further categorised stressors into key domains including economic stressors and social stressors. Here, social stressors reflected difficulties impacting one’s social life (such as discrimination and difficulties engaging in leisure activities or cultural practices) and economic stressors encompassed financial, occupational and housing difficulties. A mean score of nine items indexing social stressors and a separate mean of seven items indexing economic stressors were computed for each timepoint. To verify this categorisation, we conducted a confirmatory factor analysis, which yielded good model fit indices: RMSEA = 0.071 (95% CI = 0.066, 0.076), CFI = 0.915, TLI = 0.895, SRMR = .053. These items are presented in Table 1. Each stressor was rated on a 5-point scale (1 = *was not a problem/did not happen*, 5 = *a very serious problem*). Internal consistency was excellent across all timepoints (social stressors: *α*’s = .84 to .88, economic stressors: *α*’s = .84 to .87).

**Table 1.** Participant characteristics at baseline

Sample characteristics	Mean/*N*	SD/%
Age (yrs)	30.53	9.07
Gender (Female)	348	28.00%
Language		
Arabic	371	29.80%
English	216	17.50%
Farsi	226	18.20%
Somali	162	13.00%
Dari	260	20.90%
Country of birth		
Afghanistan	440	35.69%
Iraq	259	21.01%
Somalia	187	15.17%
Iran	92	7.46%
Sudan	73	5.92%
Pakistan	49	3.97%
Other	133	10.78%
Education		
Little or no formal education	162	13.20%
Completed primary school	374	30.48%
Completed high school	424	34.56%
Completed university	178	14.51%
Completed other training	89	7.25%
Marital status		
Married or in a relationship	565	45.97%
Not in a relationship	664	54.03%
Time in Indonesia (yrs)	5.10	1.62
Exposure to potentially traumatic events (PTEs)	7.75	5.02
Mental health		
PTSD symptom severity	0.97	0.76
Depression symptom severity	1.32	0.81
Probable PTSD diagnosis	457	46.4%
Probable depression diagnosis	644	55.1%
***Social stressors***		
1. Difficulties communicating with other people (e.g., due to language barriers)	2.29	1.17
2. Conflict with other people	1.76	1.10
3. Discrimination	2.27	1.38
4. Difficulty feeling accepted by my local community because of racial or religious differences	2.18	1.37
5. Separation from your family	2.77	1.66
6. Difficulties accessing or engaging in leisure activities (e.g., sport, cultural activities, reading, listening to music)	2.65	1.46
7. Difficulties engaging in important social activities (e.g., visiting or spending time with friends or family)	2.60	1.48
8. Difficulties engaging in cultural practices (e.g., performing religious or cultural ceremonies, obtaining culturally appropriate food, etc.)	2.28	1.41
9. Loneliness or isolation	3.14	1.53
***Economic stressors***		
1. Meeting financial obligations to family back home?	3.11	1.62
2. Difficulties related to work (e.g., not being allowed to work, not being able to find work, bad working conditions)?	4.00	1.31
3. Difficulties with accessing or undertaking study?	3.77	1.37
4. Not having enough money to meet your daily needs (e.g., for food, rent, bills, clothing or transport)?	3.77	1.26
5. Difficulty accessing urgent support from NGOs or charities (such as food or cash assistance)?	3.27	1.44
6. Difficulties accessing transport?	2.41	1.41
7. Difficulties related to housing (e.g., safety, understanding IOM housing rules, poor housing conditions, difficulties finding somewhere suitable to live, having to frequently change your place of residence)?	2.77	1.51

*Note: N* = 1,235. PTE exposure ranged from 0 to 19. PTSD symptom severity ranged from 0 to 3. Depression symptom severity ranged from 1 to 4. Economic and social stressors ranged from 1 (*was not a problem/did not happen*) to 5 (*a very serious problem*).

**Emotion dysregulation.** An 18-item version of the Difficulties in Emotion Regulation Scale (DERS; Gratz and Roemer, 2004) was used to measure emotion dysregulation.^3^ A total score was computed for each timepoint, where higher scores indicated greater emotion dysregulation. Internal consistency was excellent across all timepoints (*α*’s = .95 to .97).

### Procedure

Baseline data (T1) were collected between February and October 2020. Following timepoints (T2–T4) were then administered at 6-month intervals. This meant that the current study took place during the COVID-19 pandemic, a period of heightened economic and social stressors for refugees in Indonesia, which allowed us to uniquely capture the impact of this context. Participants completed the online survey in their chosen language via a personalised KeySurvey link. Participants received an IDR100,000 ($USD7) shopping voucher upon completing each timepoint. Ethics approval was provided by the University of New South Wales Human Research Ethics Committee (HC190494) and Atma Jaya University, Jakarta (0792/III/LPPM-PM.10.05/07/2019).

### Data analysis

**Figure 1.**
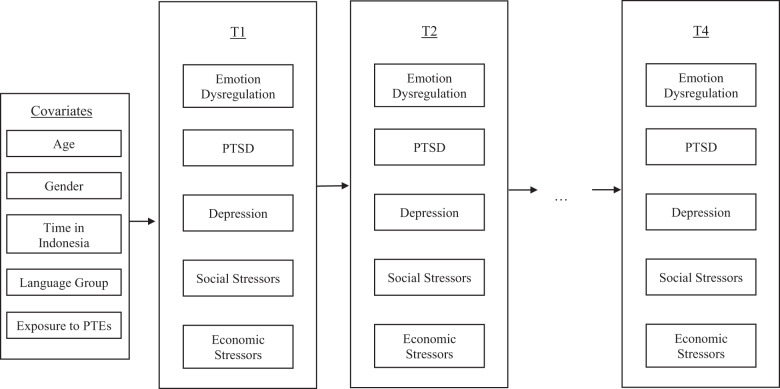
Schematic representation of variables used in the random intercept cross-lagged model (RI-CLPM). Note that while observed scores are illustrated above, RI-CLPM generates latent variables of T1-T5 observed scores (including between-persons random intercepts and within-persons deviations). PTEs = Potentially traumatic events.

To investigate the within-persons temporal relationships and between-persons associations, random intercept cross-lagged panel analysis (Hamaker *et al.*, 2015) was conducted using Mplus Version 8. Figure 1 provides a schematic representation of the variables included in the present random-intercept cross-lagged panel model (RI-CLPM). Random intercepts (RI.PTSD, RI.Depression, RI.EmDysreg, RI.Social and RI.Economic) were specified for each variable (PTSD_1_-PTSD_4_, Depression_1_-Depression_4_, EmDysreg_1_-EmDysreg_4_, Social_1_-Social_4_ and Economic_1_-Economic_4_) to capture stable (time-invariant) differences between participants across these variables. Within-persons components (wPTSD_1_-wPTSD_4_, wDepression_1_-wDepression_4_, wEmDysreg_1_-wEmDysreg_4_, wSocial_1_-wSocial_4_ and wEconomic_1_-wEconomic_4_) were specified to capture the differences between a participant’s observed measurement and their expected score (derived from the grand mean and random intercepts) for each variable, at a given timepoint. The within-persons portion of the RI-CLPM estimates included both autoregressive effects (to test that persistence of each variable overtime) and cross-lagged effects (to determine whether one’s level of a particular variable at one timepoint predicts changes in the other variables at the next timepoint).

RI-CLPM specifications followed that of Hamaker *et al.* (2015). Full information maximum likelihood estimation was used to handle missing data. Autoregressive and cross-lagged paths were constrained to equality. This approach was informed by three factors: (1) the time interval between each measurement occasion were equal (6 months), (2) we did not expect to observe systematic differences between the timepoints and (3) constraining pathways is recommended for complex models to increase their stability and interpretability. Model fit was evaluated according to the following conventions: CFI > 0.95, TLI > 0.95, SRMR < 0.08 and RMSEA < 0.06 (Hu and Bentler, 1999). As a final step, age, gender, time in Indonesia, exposure to PTEs and language group were included as covariates. Continuous covariates were mean centred. Missing data rates across all covariates were low (0.7–1%), except for PTE exposure (18% missing). Accordingly, multiple imputation (20 datasets) was used to account for missing data on the PTE exposure variable.

## Results

### Sample characteristics

Demographic characteristics of the sample are summarised in Table 1. Participants had a mean age of 30.53 years (*SD* = 9.07) and had been living in Indonesia for an average of 5.1 years (*SD* = 1.62). The majority of participants were male and originated from Afghanistan or Iraq. Participants had been exposed to a mean of 7.75 different PTEs. Rates of probable PTSD and depression in our sample were high (PTSD = 46.4%, depression = 55.1%).

### Preliminary analyses

The correlations between PTS, depression, economic and social stressors, and emotion dysregulation for each timepoint are presented in supplemental Table A. All variables yielded significant and positive bivariate correlations at each timepoint.

### Random intercept cross-lagged panel analysis

*Model fit.* Goodness-of-fit indices for our RI-CLPM indicated excellent fit: CFI = .993, TLI = .989, SRMR = .034 and RMSEA = .018 (90% confidence interval = .013–.023).

*Between-persons correlations.* All between-persons components of the model (random intercepts) yielded significant variance, indicating the presence of stable between-persons differences in levels of economic and social stressors, emotion dysregulation and symptoms of PTS and depression. Additionally, all random intercepts were positively and significantly correlated (see supplemental Table B).

### Within-persons associations between PTS and depression symptoms, economic and social stressors and emotion dysregulation

**Figure 2.**
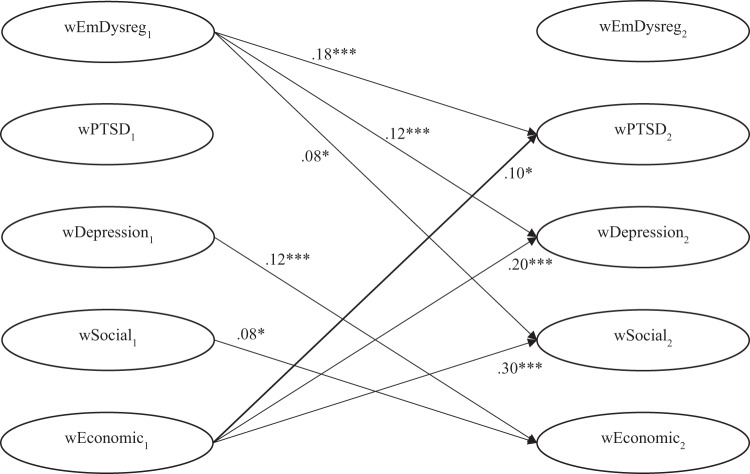
Temporal ordering of emotion dysregulation, PTSD, depression and social and economic stressors: standardized cross-lagged effects. For ease of interpretation, only the significant within-persons cross-lagged paths are shown. (As the model was constrained, estimates of the standardized cross-lagged effects from T1 to T2 are very similar to those from T2 to T3, T3 to T4 and T4 to T5.) EmDysreg = emotion dysregulation, Social = social stressors, Economic = economic stressors. *p < .05, **p < .01, ***p < .001.

The standardised and unstandardised autoregressive effects and cross-lagged effects are presented in Table 2. The correlations between the within-persons components are presented in supplemental Table B. Figure 2 shows the standardised cross-lagged effects for the overall model.

**Table 2.** Unstandardised and standardised within-persons autoregressive effects and cross-lagged paths

Variables		Unstandardised estimate (*B*)	SE	Standardised estimate range T1–T4	*p*
Within-persons autoregressive effects					
wEmDysreg_1_→	wEmDysreg_2_	0.26	0.05	0.22–0.26	<.001
wPTSD_1_→	wPTSD_2_	0.14	0.05	0.12–0.14	0.012
wDepression_1_→	wDepression_2_	0.15	0.05	0.13–0.15	0.002
wSocial_1_→	wSocial_2_	0.16	0.06	0.14–0.16	0.007
wEconomic_1_→	wEconomic_2_	0.46	0.05	0.40–0.47	<.001
Within-persons cross-lagged paths					
wEmDysreg_1_ →	wPTSD_2_	0.16	0.03	0.18–0.21	**<.001**
wEmDysreg_1_ →	wDepression_2_	0.13	0.04	0.12–0.15	**<.001**
wEmDysreg_1_ →	wSocial_2_	0.10	0.04	0.08–0.10	**0.017**
wEmDysreg_1_ →	wEconomic_2_	0.03	0.04	0.02	0.578
wPTSD_1_ →	wEmDysreg_2_	0.08	0.06	0.06–0.07	0.189
wPTSD_1_ →	wDepression_2_	0.11	0.05	0.08–0.09	**0.047**
wPTSD_1_ →	wSocial_2_	0.01	0.06	0.01	0.833
wPTSD_1_ →	wEconomic_2_	−0.12	0.06	−0.07 to −0.09	0.053
wDepression_1_ →	wEmDysreg_2_	0.08	0.05	0.06–0.07	0.141
wDepression_1_ →	wPTSD_2_	0.07	0.04	0.07–0.08	0.080
wDepression_1_ →	wSocial_2_	0.06	0.05	0.05	0.211
wDepression_1_ →	wEconomic_2_	0.18	0.05	0.12–0.14	**0.001**
wSocial_1_ →	wEmDysreg_2_	0.03	0.06	0.02–0.03	0.633
wSocial_1_ →	wPTSD_2_	0.01	0.04	0.01	0.883
wSocial_1_ →	wDepression_2_	−0.04	0.05	−0.04 to −0.05	0.370
wSocial_1_ →	wEconomic_2_	0.12	0.06	0.08–0.10	**0.037**
wEconomic_1_ →	wEmDysreg_2_	0.07	0.04	0.07–0.08	0.118
wEconomic_1_ →	wPTSD_2_	0.07	0.04	0.10	**0.047**
wEconomic_1_ →	wDepression_2_	0.17	0.04	0.20–0.22	**<.001**
wEconomic_1_ →	wSocial_2_	0.28	0.04	0.30–0.34	**<.001**

*Note:* For ease of interpretation, significant cross-lagged effects (where *p* < .05) have been bolded. EmDysreg = emotion dysregulation, Social = social stressors, Economic = economic stressors.

***The relationship between economic and social stressors and psychopathology overtime.*** We found a temporal relationship between economic stressors and both PTS and depression symptoms. Specifically, economic stressors were antecedent to PTS symptoms (i.e., within-person increases in economic stressors predicted subsequent increases in PTSD). Economic stressors were also bi-directionally associated with depression symptoms, such that higher economic stressors predicted subsequent increases in depression symptoms and higher depression symptoms predicted subsequent increases in economic stressors. Contrary to our hypothesis, a significant within-person temporal relationship between social stressors and psychopathology did not emerge.

***The relationship between emotion dysregulation and psychopathology overtime.*** As hypothesised, emotion dysregulation temporally preceded both PTS and depression symptoms overtime. Specifically, elevated levels of emotion dysregulation, relative to a participant’s typical level of emotion dysregulation, predicted greater increases in both PTS and depression symptoms at the subsequent timepoint.

***The relationship between economic and social stressors and emotion dysregulation overtime.*** Economic and social stressors evidenced a significant bi-directional relationship with each other. Additionally, emotion dysregulation was antecedent to social stressors.

### Associations with covariates

The covariate paths (i.e., predictors of stable between-persons differences in overall levels of PTS and depression symptoms, economic and social stressors, and emotion dysregulation) are presented in supplemental Table C.

## Discussion

The present study was the first, to our knowledge, to concurrently and longitudinally investigate the influence of economic, social and psychological factors on psychopathology among refugees residing in an LMIC. In support of our hypotheses, economic stressors and emotion dysregulation were both identified as salient drivers of within-person increases in PTS and depressive symptoms and social stressors. Specifically, elevations in emotion dysregulation preceded increases in PTS and depressive symptoms and social stressors overtime, while elevations in economic stressors preceded increases in PTS symptoms and were bi-directionally associated with increases in depressive symptoms and social stressors. Contrary to our hypotheses, after accounting for the impact of economic stressors and emotion dysregulation, social stressors were not directly associated with within-person changes in PTS or depressive symptoms overtime.

The first key finding was that economic stressors preceded increases in PTS symptoms and were bi-directionally associated with increases in both depression symptoms and social stressors. This supports an existing body of research highlighting the salience of economic stressors (e.g., financial hardship, poverty and unemployment) to mental and social wellbeing among refugees in high-income countries (Beiser and Hou, 2001; O’Donnell *et al.*, 2020; Torlinska *et al.*, 2020). Economic stressors may have been a particularly impactful determinant for our participants, who resided in Indonesia – an LMIC where it is illegal for refugees to work, open a bank account or hold a driver’s license (Indonesian Association for the Protection of Refugee Rights, 2023). These restrictions may not only drive economic hardship but create a stressful environment that exacerbates psychological symptoms and limits social participation. Policy changes that remove key barriers to livelihood and economic empowerment thus represent a critical pathway to promote the psychological, social and economic wellbeing of refugees in displacement settings.

Another key finding was that emotion dysregulation led to the worsening of PTS and depression symptoms as well as social stressors overtime. This finding extends prior longitudinal research, which found emotion dysregulation to be a driver of PTS symptoms in refugees resettled in a high-income country (Specker *et al.*, 2024). The present study revealed that this relationship was also evident among refugees in an LMIC and followed the same pattern for depression symptoms and social stressors. Considering this, it may be that greater difficulties in regulating one’s emotions, especially in a transit setting where individuals contend with numerous and unrelenting stressors, can deplete emotional resources and undermine both social participation and psychological wellbeing. Taken together, our findings provide support for the robustness of emotion dysregulation as an important transdiagnostic factor underpinning both psychological and social difficulties amongst displaced refugees. Furthermore, these findings point to the utility of interventions that target emotion dysregulation as a way to bring about improvements in both psychological and social wellbeing for refugees in displacement settings.

It was noteworthy that our study did not find evidence of a longitudinal within-person relationship between social stressors and psychopathology. This finding is somewhat surprising given extensive past research supporting a link between social difficulties and poorer mental health among refugees (Schick *et al.*, 2016; Berthold *et al.*, 2019). A reason for this apparent discrepancy may be due to methodological differences between our study and past research. Prior studies have predominantly been cross-sectional and, thus, explored between-person effects. Our findings are consistent with these prior results such that we also found a significant between-person effect; refugees with higher overall levels of social stressors also exhibited elevated PTS and depression symptoms, emotion dysregulation and economic stressors (represented by significant associations between random intercepts). However, when investigating within-person effects, individual changes in social stressors did not significantly influence subsequent changes in PTS or depression symptoms for individuals in our sample. Much of the past literature has explored the impact of social factors on mental health in isolation and in high-income countries. Given the interrelated nature between social, economic and psychological functioning, it may be that economic stressors are more salient for refugees in LMIC transitory settings where legal and economic rights are not guaranteed by the host country.

### Limitations

Several study limitations merit acknowledgment. First, while the current study included a large and diverse cohort of refugees displaced in Indonesia, there is substantial variability in the living conditions between different LMICs due to differing domestic policies and availability of essential services. Consequently, it is unclear how our findings may generalise to other displaced populations in different settings. Further research across countries is needed to verify which factors or relationships are universal and which are country-specific. Second, the current study operationalised economic, social and psychological factors by indexing economic stressors, social stressors and emotion dysregulation. While each of these variables was derived from established evidence-bases, it is noteworthy that other relevant factors exist. For example, regarding psychological factors, intolerance of uncertainty (Nickerson *et al.*, 2023b) and psychological flexibility (Lakin *et al.*, 2023) may be relevant given the dynamic and uncontrollable nature of transit environments. Regarding economic and social stressors, these were measured via self-report measures. While these items have been well-validated, future research could improve upon this by also including objective markers of employment, sources of income, housing situation, social networks, and the frequency and quality of social interactions or support. It is also noteworthy that the present study comprised a culturally diverse sample of refugees with differing experiences and levels of engagement. For example, Farsi- and Dari-speaking participants had greater retention across our study timepoints than Arabic-speaking participants. Although beyond the scope of the present study due to sample size constraints, it would be valuable for future research to explore whether the relationships between environmental stressors, emotion dysregulation and mental health differ between these cultural groups. Lastly, as the current study was conducted during the COVID-19 pandemic, which was a time of heightened environmental stress for refugees, we cannot verify how these factors compare to other time periods. Future studies might benefit from examining changes in contextual stressors before and after major events or policy changes to better measure their impact on mental health over time.

## Conclusions

Notwithstanding these limitations, our findings provide insights that may be useful to inform service provision and policy for refugees in transit contexts. First, they highlight a need to ameliorate the uncontrollable stressors faced by refugees in displacement, such as denial of refugee status and work rights. Research from high-income countries has highlighted the positive impact of increased economic empowerment on refugee mental health. For example, studies found that a refugee’s ability to obtain employment reduced financial hardship and improved health overtime (Torlinska *et al.*, 2020) and that the provision of permanent visa status, which granted work rights, led to subsequent reductions in psychopathology (Nickerson *et al.*, 2023a). Improvements in the legal and economic status of refugees are thus critically important. Alongside this, and especially as structural and policy reform can be slow to occur, complementary interventions that strengthen refugees’ psychological coping capacities may be useful. Given the salience of emotion dysregulation to the exacerbation of psychopathology and social stressors demonstrated in this study, psychological interventions that enhance skills in emotion-focused coping may also be an effective way to improve wellbeing. Indeed, scalable emotion-focused coping interventions are showing promising results among refugee populations living in resource-scarce settings (Acarturk *et al.*, 2022). Taken together, our findings support the need for both policy changes that directly address barriers to economic participation as well as resourcing of interventions that support emotion-focused coping in these settings. Thus, multi-level interventions combining opportunities for economic empowerment (e.g., skills training, financial assistance) and psychological programs may offer more effective solutions to enhance social cohesion and mitigate the long-term psychological impacts of living with uncertainty and daily stressors (Miller and Rasmussen, 2024).

## Supporting information

10.1017/S2045796026100493.sm001 Specker et al. supplementary material Specker et al. supplementary material

## Data Availability

The dataset is available from the corresponding author on reasonable request. Analysis code has been provided in the supplemental materials.

## Footnotes

1 For simplicity, we use the term ‘refugee’ throughout this paper to refer to a subset of the global forcibly displaced population that have crossed international borders.

2 Participants who completed all timepoints had lived in Indonesia for longer than those who did not (mean = 5.37 years vs 4.84 years, *t*(1230) = 5.76, *p* < 0.001). While there was no difference between the two groups in the proportion of participants who completed the survey in Somali (all timepoints = 11.36% vs not all timepoints = 14.48%), compared to Arabic (all timepoints = 27.76% vs not all timepoints = 2.94%, *χ^2^*(1) = 2.68, *p* = 0.107), participants who completed all timepoints were more likely to have undertaken the survey in Dari (all timepoints = 24.6% vs not all timepoints = 17.30%, *χ^2^*(1) = 9.90, *p* = 0.002) or Farsi (all timepoints = 22.08% vs not all timepoints = 14.31%, *χ^2^*(1) = 12.47, *p* < 0.001) but less likely to have undertaken the survey in English (all timepoints = 14.20% vs not all timepoints = 20.97%, *χ^2^*(1) = 9.80, *p* = 0.002).

3 We used an adapted version of the scale, where three of the original items that originally required reverse scoring were re-worded to ensure all items were valanced in the same direction. Specifically, ‘*I pay attention to how I feel*’ was reworded to ‘*I do not pay attention to how I feel*’, ‘*I am attentive to my feelings*’ was reworded to ‘*I am not attentive to my feelings*’ and ‘*When I am upset, I acknowledge my emotions*’ was reworded to ‘*I do not acknowledge my emotions when I am upset*’. This decision was informed by prior cross-cultural research demonstrating poor internal consistency among reverse-scored items (Nickerson *et al.*, 2015; Schlechter *et al.*, 2021). This adapted scale has previously been validated and used with refugees (Doolan *et al.*, 2017; Specker *et al.*, 2024).

## Supplementary material

The supplementary material for this article can be found at https://doi.org/10.1017/S2045796026100493.
