# Supplementary material for: The longitudinal relationship between economic and social stressors, emotion dysregulation and mental health among refugees in protracted displacement
Source: Epidemiol Psychiatr Sci. 2026 Mar 27;35:e14. doi: 10.1017/S2045796026100493 (PMC13122541; doi:10.1017/S2045796026100493)
Supplement: Specker et al. supplementary material [file S2045796026100493sup001.docx]

The longitudinal relationship between emotion dysregulation, social and economic stressors, and psychopathology among refugees in protracted displacement

Philippa Specker PhD^1^, Gulsah Kurt PhD^1^, Belinda Liddell PhD^1,2^, David Keegan^3^, Randy Nandyatama PhD^4^, Atika Yuanita^5^, Rizka Argadianti Rachmah^1^, Joel Hoffman PhD^1^, Shraddha Kashyap PhD^7^, Diah Tricesaria^6^, Mitra Khakbaz^3^, Zico Pestalozzi^5^, & Angela Nickerson PhD^1^

^1^School of Psychology, University of New South Wales, Sydney, Australia

^2^School of Psychological Sciences, University of Newcastle, Australia

^3^HOST International, 31 Grose Street, Parramatta, NSW, Australia

^4^Department of International Relations, Gadjah Mada University Yogyakarta, Bulaksumur, Yogyakarta 55281, Indonesia

^5^SUAKA, Indonesian Civil Society Network for Refugee Rights Protection, Menteng, Jakarta Pusat 10320, Indonesia

^6^School of Social Sciences, Monash University, Melbourne, Australia

^7^Bilya Marlee School of Indigenous Studies, University of Western Australia, Perth, Australia

**Supplementary Online Material**

**Supplemental Table A.** Correlations between emotion dysregulation, social and economic stressors, PTSD and depression.

**Supplemental Table B.** Random intercept cross-lagged model: Random intercepts and within-persons components (correlations and variance).

**Supplemental Table C.** Random intercept cross-lagged model: Covariate predictors.

**Analysis Code.** MPlus syntax used for main analysis (random intercept cross-lagged panel analysis).

**Supplemental Table A.** Correlations for Emotion dysregulation, PTSD, Depression and Social and Economic Stressors T1 to T4.

|  | T1 ED | T2 ED | T3 ED | T4 ED | T1 PTSD | T2 PTSD | T3 PTSD | T4 PTSD | T1 Dep | T2 Dep | T3 Dep | T4 Dep | T1 Soc | T2 Soc | T3 Soc | T4 Soc | T1 Eco | T2 Eco | T3 Eco | T4 Eco |
| --- | --- | --- | --- | --- | --- | --- | --- | --- | --- | --- | --- | --- | --- | --- | --- | --- | --- | --- | --- | --- |
| T1 ED | -- |  |  |  |  |  |  |  |  |  |  |  |  |  |  |  |  |  |  |  |
| T2 ED | 0.62 | -- |  |  |  |  |  |  |  |  |  |  |  |  |  |  |  |  |  |  |
| T3 ED | 0.59 | 0.69 | -- |  |  |  |  |  |  |  |  |  |  |  |  |  |  |  |  |  |
| T4 ED | 0.55 | 0.63 | 0.70 | -- |  |  |  |  |  |  |  |  |  |  |  |  |  |  |  |  |
| T1 PTSD | 0.67 | 0.51 | 0.53 | 0.48 | -- |  |  |  |  |  |  |  |  |  |  |  |  |  |  |  |
| T2 PTSD | 0.53 | 0.69 | 0.57 | 0.54 | 0.66 | -- |  |  |  |  |  |  |  |  |  |  |  |  |  |  |
| T3 PTSD | 0.52 | 0.64 | 0.72 | 0.62 | 0.61 | 0.70 | -- |  |  |  |  |  |  |  |  |  |  |  |  |  |
| T4 PTSD | 0.51 | 0.54 | 0.65 | 0.73 | 0.59 | 0.67 | 0.71 | -- |  |  |  |  |  |  |  |  |  |  |  |  |
| T1 Dep | 0.65 | 0.51 | 0.49 | 0.44 | 0.74 | 0.53 | 0.53 | 0.48 | -- |  |  |  |  |  |  |  |  |  |  |  |
| T2 Dep | 0.53 | 0.70 | 0.55 | 0.55 | 0.54 | 0.75 | 0.62 | 0.58 | 0.60 | -- |  |  |  |  |  |  |  |  |  |  |
| T3 Dep | 0.54 | 0.62 | 0.74 | 0.63 | 0.55 | 0.64 | 0.80 | 0.68 | 0.57 | 0.68 | -- |  |  |  |  |  |  |  |  |  |
| T4 Dep | 0.53 | 0.54 | 0.60 | 0.72 | 0.50 | 0.57 | 0.62 | 0.82 | 0.54 | 0.62 | 0.70 | -- |  |  |  |  |  |  |  |  |
| T1 Soc | 0.52 | 0.48 | 0.47 | 0.45 | 0.63 | 0.53 | 0.53 | 0.50 | 0.61 | 0.52 | 0.53 | 0.48 | -- |  |  |  |  |  |  |  |
| T2 Soc | 0.39 | 0.52 | 0.46 | 0.42 | 0.48 | 0.57 | 0.56 | 0.46 | 0.46 | 0.59 | 0.52 | 0.46 | 0.65 | -- |  |  |  |  |  |  |
| T3 Soc | 0.43 | 0.52 | 0.57 | 0.50 | 0.52 | 0.60 | 0.65 | 0.56 | 0.49 | 0.57 | 0.65 | 0.55 | 0.64 | 0.73 | -- |  |  |  |  |  |
| T4 Soc | 0.47 | 0.52 | 0.58 | 0.58 | 0.50 | 0.56 | 0.55 | 0.65 | 0.50 | 0.56 | 0.60 | 0.63 | 0.61 | 0.68 | 0.76 | -- |  |  |  |  |
| T1 Eco | 0.37 | 0.37 | 0.41 | 0.30 | 0.46 | 0.43 | 0.46 | 0.39 | 0.46 | 0.45 | 0.42 | 0.37 | 0.71 | 0.59 | 0.57 | 0.51 | -- |  |  |  |
| T2 Eco | 0.29 | 0.37 | 0.39 | 0.36 | 0.35 | 0.43 | 0.48 | 0.40 | 0.39 | 0.50 | 0.48 | 0.40 | 0.53 | 0.71 | 0.63 | 0.55 | 0.67 | -- |  |  |
| T3 Eco | 0.33 | 0.41 | 0.45 | 0.41 | 0.40 | 0.47 | 0.52 | 0.45 | 0.40 | 0.48 | 0.51 | 0.45 | 0.49 | 0.60 | 0.74 | 0.59 | 0.60 | 0.74 | -- |  |
| T4 Eco | 0.35 | 0.38 | 0.42 | 0.45 | 0.39 | 0.44 | 0.45 | 0.51 | 0.39 | 0.44 | 0.49 | 0.55 | 0.48 | 0.56 | 0.63 | 0.75 | 0.52 | 0.66 | 0.72 | -- |

Note: All correlations were significant at *p* < .001. T = timepoint, ED = emotion dysregulation, PTSD = Posttraumatic Stress Disorder, Dep = Depression, Soc = social stressors, Eco = economic stressors.

**Supplemental Table B.** Random intercept cross-lagged model: Random intercepts and within-persons components (correlations and variance).

| Correlations | | Unstandardized estimate (B) | S.E. | Standardized estimate | *p* |
| --- | --- | --- | --- | --- | --- |
| ***Random Intercepts*** | |  |  |  |  |
| RI.EmDysreg with | RI.PTSD | 0.18 | 0.02 | 0.76 | <.001 |
|  | RI.Depression | 0.18 | 0.02 | 0.78 | <.001 |
|  | RI.Social | 0.13 | 0.03 | 0.52 | <.001 |
|  | RI.Economic | 0.07 | 0.03 | 0.29 | 0.015 |
| RI.PTSD with | RI.Depression | 0.17 | 0.02 | 0.81 | <.001 |
|  | RI.Social | 0.14 | 0.02 | 0.62 | <.001 |
|  | RI.Economic | 0.08 | 0.03 | 0.37 | 0.002 |
| RI.Depression with | RI.Social | 0.14 | 0.02 | 0.65 | <.001 |
|  | RI.Economic | 0.09 | 0.03 | 0.39 | 0.003 |
| RI.Social with | RI.Economic | 0.14 | 0.04 | 0.59 | 0.001 |
| ***Within-person components*** | |  |  |  |  |
| T1 | |  |  |  |  |
| wEmDysreg with | wPTSD | 0.15 | 0.02 | 0.50 | <.001 |
|  | wDepression | 0.15 | 0.02 | 0.44 | <.001 |
|  | wSocial | 0.10 | 0.03 | 0.28 | <.001 |
|  | wEconomic | 0.09 | 0.03 | 0.20 | 0.004 |
| wPTSD with | wDepression | 0.18 | 0.02 | 0.64 | <.001 |
|  | wSocial | 0.12 | 0.02 | 0.39 | <.001 |
|  | wEconomic | 0.11 | 0.03 | 0.28 | <.001 |
| wDepression with | wSocial | 0.13 | 0.02 | 0.37 | <.001 |
|  | wEconomic | 0.13 | 0.03 | 0.30 | <.001 |
| wSocial with | wEconomic | 0.30 | 0.04 | 0.63 | <.001 |
| T2 | |  |  |  |  |
| wEmDysreg with | wPTSD | 0.20 | 0.02 | 0.57 | <.001 |
|  | wDepression | 0.23 | 0.02 | 0.56 | <.001 |
|  | wSocial | 0.15 | 0.02 | 0.36 | <.001 |
|  | wEconomic | 0.13 | 0.02 | 0.25 | <.001 |
| wPTSD with | wDepression | 0.21 | 0.02 | 0.67 | <.001 |
|  | wSocial | 0.12 | 0.02 | 0.35 | <.001 |
|  | wEconomic | 0.11 | 0.02 | 0.28 | <.001 |
| wDepression with | wSocial | 0.16 | 0.02 | 0.40 | <.001 |
|  | wEconomic | 0.17 | 0.02 | 0.38 | <.001 |
| wSocial with | wEconomic | 0.31 | 0.03 | 0.63 | <.001 |
| T3 | |  |  |  |  |
| wEmDysreg with | wPTSD | 0.19 | 0.02 | 0.51 | <.001 |
|  | wDepression | 0.21 | 0.02 | 0.54 | <.001 |
|  | wSocial | 0.14 | 0.02 | 0.34 | <.001 |
|  | wEconomic | 0.12 | 0.02 | 0.24 | <.001 |
| wPTSD with | wDepression | 0.20 | 0.02 | 0.63 | <.001 |
|  | wSocial | 0.13 | 0.02 | 0.42 | <.001 |
|  | wEconomic | 0.12 | 0.02 | 0.31 | <.001 |
| wDepression with | wSocial | 0.13 | 0.02 | 0.38 | <.001 |
|  | wEconomic | 0.12 | 0.02 | 0.29 | <.001 |
| wSocial with | wEconomic | 0.25 | 0.02 | 0.62 | <.001 |
| T4 | |  |  |  |  |
| wEmDysreg with | wPTSD | 0.18 | 0.02 | 0.51 | <.001 |
|  | wDepression | 0.20 | 0.02 | 0.49 | <.001 |
|  | wSocial | 0.11 | 0.02 | 0.27 | <.001 |
|  | wEconomic | 0.11 | 0.02 | 0.24 | <.001 |
| wPTSD with | wDepression | 0.23 | 0.02 | 0.71 | <.001 |
|  | wSocial | 0.13 | 0.02 | 0.41 | <.001 |
|  | wEconomic | 0.11 | 0.02 | 0.32 | <.001 |
| wDepression with | wSocial | 0.13 | 0.02 | 0.34 | <.001 |
|  | wEconomic | 0.15 | 0.02 | 0.37 | <.001 |
| wSocial with | wEconomic | 0.27 | 0.02 | 0.64 | <.001 |
|  |  |  |  |  |  |
| ***Variance*** | | Unstandardized estimate (B) | S.E. |  | *p* |
| wEmDysreg_1_ |  | 0.37 | 0.03 |  | <.001 |
| wPTSD_1_ |  | 0.25 | 0.02 |  | <.001 |
| wDepression_1_ |  | 0.33 | 0.02 |  | <.001 |
| wSocial_1_ |  | 0.38 | 0.04 |  | <.001 |
| wEconomic_1_ |  | 0.59 | 0.06 |  | <.001 |
|  | |  |  |  |  |
| ***Residual Variance*** | | Unstandardized estimate (B) | S.E. | Standardized estimate | *p* |
| RI.EmDysreg |  | 0.27 | 0.03 | 0.64 | <.001 |
| RI.PTSD |  | 0.21 | 0.02 | 0.64 | <.001 |
| RI.Depression |  | 0.21 | 0.02 | 0.62 | <.001 |
| RI.Social |  | 0.23 | 0.04 | 0.52 | <.001 |
| RI.Economic |  | 0.23 | 0.06 | 0.55 | <.001 |
| wEmDysreg2 |  | 0.45 | 0.03 | 0.89 | <.001 |
| wEmDysreg3 |  | 0.47 | 0.03 | 0.85 | <.001 |
| wEmDysreg4 |  | 0.46 | 0.03 | 0.84 | <.001 |
| wPTSD2 |  | 0.26 | 0.02 | 0.87 | <.001 |
| wPTSD3 |  | 0.29 | 0.02 | 0.84 | <.001 |
| wPTSD4 |  | 0.28 | 0.02 | 0.82 | <.001 |
| wDepression2 |  | 0.37 | 0.02 | 0.87 | <.001 |
| wDepression3 |  | 0.33 | 0.02 | 0.80 | <.001 |
| wDepression4 |  | 0.36 | 0.02 | 0.80 | <.001 |
| wSocial2 |  | 0.41 | 0.03 | 0.80 | <.001 |
| wSocial3 |  | 0.34 | 0.02 | 0.70 | <.001 |
| wSocial4 |  | 0.38 | 0.03 | 0.72 | <.001 |
| wEconomic2 |  | 0.58 | 0.04 | 0.76 | <.001 |
| wEconomic3 |  | 0.49 | 0.03 | 0.66 | <.001 |
| wEconomic4 |  | 0.45 | 0.03 | 0.65 | <.001 |

Note: Subscript number denotes time point. RI = random intercept, EmDysreg = emotion dysregulation, Social = social stressors, Economic = economic stressors, w = within-persons component.

**Supplemental Table C.** Random intercept cross-lagged model: Covariate predictors.

|  | | B | | S.E. | | Standardised estimate | | *p* | |  |
| --- | --- | --- | --- | --- | --- | --- | --- | --- | --- | --- |
| Age → | RI.EmDysreg | | 0.00 | | 0.00 | | 0.02 | | 0.605 | |
|  | RI.PTSD | | 0.00 | | 0.00 | | 0.02 | | 0.514 | |
|  | RI.Depression | | 0.00 | | 0.00 | | -0.01 | | 0.819 | |
|  | RI.Social | | 0.00 | | 0.00 | | 0.00 | | 0.956 | |
|  | RI.Economic | | 0.00 | | 0.00 | | 0.01 | | 0.869 | |
| Gender → | RI.EmDysreg | | 0.08 | | 0.05 | | 0.06 | | 0.099 | |
|  | RI.PTSD | | 0.06 | | 0.04 | | 0.05 | | 0.124 | |
|  | RI.Depression | | 0.07 | | 0.04 | | 0.05 | | 0.125 | |
|  | RI.Social | | 0.05 | | 0.05 | | 0.04 | | 0.287 | |
|  | RI.Economic | | -0.03 | | 0.06 | | -0.02 | | 0.537 | |
| Time in Indonesia → | RI.EmDysreg | | -0.02 | | 0.01 | | -0.06 | | 0.111 | |
|  | RI.PTSD | | -0.02 | | 0.01 | | -0.06 | | 0.061 | |
|  | RI.Depression | | 0.02 | | 0.01 | | 0.05 | | 0.150 | |
|  | RI.Social | | 0.02 | | 0.01 | | 0.06 | | 0.103 | |
|  | RI.Economic | | 0.05 | | 0.02 | | 0.14 | | 0.001 | |
| Exposure to PTEs → | RI.EmDysreg | | 0.05 | | 0.00 | | 0.40 | | <.001 | |
|  | RI.PTSD | | 0.06 | | 0.00 | | 0.50 | | <.001 | |
|  | RI.Depression | | 0.05 | | 0.00 | | 0.43 | | <.001 | |
|  | RI.Social | | 0.07 | | 0.00 | | 0.55 | | <.001 | |
|  | RI.Economic | | 0.07 | | 0.01 | | 0.57 | | <.001 | |
| English vs Arabic Language → | RI.EmDysreg | | 0.49 | | 0.07 | | 0.29 | | <.001 | |
|  | RI.PTSD | | 0.44 | | 0.06 | | 0.30 | | <.001 | |
|  | RI.Depression | | 0.40 | | 0.06 | | 0.26 | | <.001 | |
|  | RI.Social | | 0.47 | | 0.07 | | 0.27 | | <.001 | |
|  | RI.Economic | | 0.27 | | 0.08 | | 0.16 | | <.001 | |
| Farsi vs Arabic Language → | RI.EmDysreg | | 0.53 | | 0.06 | | 0.32 | | <.001 | |
|  | RI.PTSD | | 0.21 | | 0.05 | | 0.15 | | <.001 | |
|  | RI.Depression | | 0.53 | | 0.06 | | 0.35 | | <.001 | |
|  | RI.Social | | 0.33 | | 0.06 | | 0.19 | | <.001 | |
|  | RI.Economic | | 0.19 | | 0.07 | | 0.11 | | 0.010 | |
| Somali vs Arabic Language → | RI.EmDysreg | | -0.16 | | 0.08 | | -0.08 | | 0.038 | |
|  | RI.PTSD | | -0.04 | | 0.07 | | -0.02 | | 0.528 | |
|  | RI.Depression | | 0.00 | | 0.07 | | 0.00 | | 0.972 | |
|  | RI.Social | | -0.27 | | 0.08 | | -0.14 | | <.001 | |
|  | RI.Economic | | 0.16 | | 0.09 | | 0.08 | | 0.082 | |
| Dari vs Arabic Language → | RI.EmDysreg | | 0.51 | | 0.06 | | 0.32 | | <.001 | |
|  | RI.PTSD | | 0.29 | | 0.05 | | 0.21 | | <.001 | |
|  | RI.Depression | | 0.47 | | 0.05 | | 0.33 | | <.001 | |
|  | RI.Social | | 0.46 | | 0.06 | | 0.28 | | <.001 | |
|  | RI.Economic | | 0.39 | | 0.07 | | 0.25 | | <.001 | |

Note: RI = random intercept, EmDysreg = emotion dysregulation, Social = social stressors, Economic = economic stressors.

**Analysis Code.** MPlus syntax used for main analysis (random intercept cross-lagged panel analysis).

*!Annotation note:*

*!x variables = Emotion Dysregulation (T1-T4)*

*!y variables = Psychopathology*

*!!ya = PTSD (T1-T4)*

*!!yb = Dep (T1-T4)*

*!z variables = Environmental Stressors*

*!!za = Social stressors (T1-T4)*

*!!zb = Economic stressors (T1-T4)*

USEVARIABLES ARE

x1 x2 x3 x4

ya1 ya2 ya3 ya4

yb1 yb2 yb3 yb4

!yc1 yc2 yc3 yc4

za1 za2 za3 za4

zb1 zb2 zb3 zb4

ageC gender timeC traumaC

English Farsi Somali Dari;

MISSING = *;

ANALYSIS:

MODEL = NOCOV; ! Sets all default covariances to zeroMODEL:

! Create between components (random intercepts)

RIx BY x1@1 x2@1 x3@1 x4@1;

RIya BY ya1@1 ya2@1 ya3@1 ya4@1;

RIyb BY yb1@1 yb2@1 yb3@1 yb4@1;

RIza BY za1@1 za2@1 za3@1 za4@1;

RIzb BY zb1@1 zb2@1 zb3@1 zb4@1;

! Create within-person centered variables

wx1 BY x1@1;

wx2 BY x2@1;

wx3 BY x3@1;

wx4 BY x4@1;

wya1 BY ya1@1;

wya2 BY ya2@1;

wya3 BY ya3@1;

wya4 BY ya4@1;

wyb1 BY yb1@1;

wyb2 BY yb2@1;

wyb3 BY yb3@1;

wyb4 BY yb4@1;

wza1 BY za1@1;

wza2 BY za2@1;

wza3 BY za3@1;

wza4 BY za4@1;

wzb1 BY zb1@1;

wzb2 BY zb2@1;

wzb3 BY zb3@1;

wzb4 BY zb4@1;

! Constrain measurement error variances to 0

x1-zb4@0;

! Estimate lagged effects between within-person centered variables & constrain to equality

wx2 ON wx1 wya1 wyb1 wza1 wzb1 (a b c d e );

wya2 ON wx1 wya1 wyb1 wza1 wzb1 (g h i j k );

wyb2 ON wx1 wya1 wyb1 wza1 wzb1 (m n o p q );

wza2 ON wx1 wya1 wyb1 wza1 wzb1 (y z aa ab ac );

wzb2 ON wx1 wya1 wyb1 wza1 wzb1 (ae af ag ah ai );

wx3 ON wx2 wya2 wyb2 wza2 wzb2 (a b c d e );

wya3 ON wx2 wya2 wyb2 wza2 wzb2 (g h i j k );

wyb3 ON wx2 wya2 wyb2 wza2 wzb2 (m n o p q );

wza3 ON wx2 wya2 wyb2 wza2 wzb2 (y z aa ab ac );

wzb3 ON wx2 wya2 wyb2 wza2 wzb2 (ae af ag ah ai );

wx4 ON wx3 wya3 wyb3 wza3 wzb3 (a b c d e );

wya4 ON wx3 wya3 wyb3 wza3 wzb3 (g h i j k );

wyb4 ON wx3 wya3 wyb3 wza3 wzb3 (m n o p q );

wza4 ON wx3 wya3 wyb3 wza3 wzb3 (y z aa ab ac );

wzb4 ON wx3 wya3 wyb3 wza3 wzb3 (ae af ag ah ai );

! Estimate covariance between random intercepts

RIx WITH RIya RIyb RIza RIzb;

RIya WITH RIyb RIza RIzb;

RIyb WITH RIza RIzb;

RIza WITH RIzb;

! Estimate covariance between within-person components at first wave

wx1 WITH wya1 wyb1 wza1 wzb1;

wya1 WITH wyb1 wza1 wzb1;

wyb1 WITH wza1 wzb1;

wza1 WITH wzb1;

! Estimate covariances between residuals of w/in components (i.e., innovations)

wx2 WITH wya2 wyb2 wza2 wzb2;

wya2 WITH wyb2 wza2 wzb2;

wyb2 WITH wza2 wzb2;

wza2 WITH wzb2;

wx3 WITH wya3 wyb3 wza3 wzb3;

wya3 WITH wyb3 wza3 wzb3;

wyb3 WITH wza3 wzb3;

wza3 WITH wzb3;

wx4 WITH wya4 wyb4 wza4 wzb4;

wya4 WITH wyb4 wza4 wzb4;

wyb4 WITH wza4 wzb4;

wza4 WITH wzb4;

!covariates

RIx RIya RIyb RIza RIzb on ageC gender timeC traumaC English Farsi Somali Dari;

OUTPUT: TECH1 STDYX SAMPSTAT CINTERVAL;
